# Body–Self Relations, Eating Behaviors, and Occupational Factors Associated with BMI and Obesity in Mass Catering Workers: A Cross-Sectional Study

**DOI:** 10.3390/healthcare14193340

**Published:** 2026-10-07

**Authors:** Hatice Baygut

**Affiliations:** 1 Department of Nutrition and Dietetics, Faculty of Health Sciences, Süleyman Demirel University, Isparta 32260, Türkiye; haticebaygut@sdu.edu.tr or hatice.baygut@manas.edu.kg; Tel.: +90-246-211-3269; 2 Department of Gastronomy and Culinary Arts, Faculty of Tourism, Kyrgyz-Turkish Manas University, Bishkek 720038, Kyrgyzstan

**Keywords:** mass catering workers, body mass index, obesity, body–self relations, eating behavior, uncontrolled eating, job control, occupational health

## Abstract

Background: Mass catering workers are repeatedly exposed to food during working hours while also experiencing time pressure and psychosocial job demands. This study aimed to examine associations of BMI and obesity with body–self relations, eating behaviors, and demand-control-support characteristics in mass catering workers. Methods: This cross-sectional study included 452 workers in Isparta, Türkiye. BMI was calculated from measured weight and height. Body–self relations, eating behaviors, and occupational characteristics were assessed using the MBSRQ, TFEQ-R18, and Swedish Demand-Control-Support Questionnaire. BMI-group comparisons, Spearman correlations, and multivariable linear regression with HC3 robust standard errors were performed; obesity models were secondary. Results: Mean BMI was 28.38 ± 4.66 kg/m^2^; 31.4% were classified as overweight and 44.2% as having obesity. body–self relation scores decreased and uncontrolled eating increased across BMI groups (both BH-adjusted *p* < 0.001). BMI correlated negatively with body–self relations (ρ = −0.465) and positively with uncontrolled eating (ρ = 0.427). In the fully adjusted model, body–self relations were inversely associated with BMI (β = −2.816, 95% CI −3.855 to −1.778), whereas uncontrolled eating was positively associated with BMI (β = 0.0347 per point, 95% CI 0.0190 to 0.0503). Conclusions: BMI variation in this workforce was characterized most consistently by body–self relations and uncontrolled eating, alongside smaller occupational associations.

## 1. Introduction

Obesity has become a dominant component of the global double burden of malnutrition. A pooled analysis of 3663 population-representative studies showed a marked worldwide shift toward obesity between 1990 and 2022, establishing excess body weight as a central population-health challenge across adult age groups [1]. Occupational populations warrant particular attention because eating opportunities, physical demands, schedules, social norms, and psychosocial exposures are concentrated within the workplace and may accumulate over years of employment.

Food-service and mass-catering workers constitute a distinct occupational population within this wider problem. Studies in government-backed restaurants and community food services have reported substantial levels of excess weight and obesity among workers, despite employment in settings organized around meal production [2,3,4]. A nationwide study of Brazilian food handlers found excess weight in nearly 60% of participants and linked longer food-service employment with greater odds of overweight [3]. Adherence to healthy dietary guidance among food-service workers has also varied with work activity, demographic factors, and quality of life [5]. Research in catering professionals similarly indicates that repeated occupational exposure to food can coexist with elevated BMI and other health risks [6].

Mass catering is particularly relevant to obesity research because food exposure is embedded in the work itself. Food preparation, tasting, serving, proximity to meals, break schedules, work pace, and social interactions occur within the same occupational environment. This concentration of behavioral and workplace exposures provides a setting in which eating patterns and psychosocial characteristics can be examined together rather than as isolated determinants. The resulting evidence can extend obesity research toward occupational environments that organize both food access and daily eating opportunities [2,3,7].

The workplace can influence eating through more than simple food availability. Employees may consume a substantial share of daily energy during working hours, and a systematic review identified job role, social context, time constraints, and the local food environment as recurring determinants of workplace eating behavior [7]. Shift and non-standard work schedules add another layer. Meta-analytic evidence indicates that shift work is associated with a modestly higher BMI [8], while qualitative and intervention evidence among night workers describes irregular meal timing, limited healthy options, fatigue, and disrupted routines as barriers to weight management [9]. These findings illustrate how work organization can shape dietary behavior and body weight.

Psychological stress is another pathway linking the occupational environment to eating behavior. A systematic review and meta-analysis in healthy adults found that stress alters both the quantity and quality of food intake, with substantial heterogeneity across individuals and contexts [10]. Occupational evidence is less uniform. A systematic review of hospital workers found associations between occupational stress and BMI in several studies but judged the overall certainty of evidence to be limited by study quality and heterogeneity [11]. Cohort meta-analysis has likewise shown no overall association between job strain and incident obesity or weight gain [12]. The demand-control framework remains useful for separating workload, decision latitude, and social support rather than treating occupational stress as a single undifferentiated exposure [13].

Eating behavior provides a more proximal behavioral layer. Emotional eating, uncontrolled eating, and cognitive restraint represent different responses to internal and external eating cues. Emotional eating has been associated with higher weight status in meta-analytic research [14], but the strength and direction of associations differ by eating-behavior dimension. The TFEQ-R18 was developed to characterize cognitive restraint, uncontrolled eating, and emotional eating in a concise form and has demonstrated the ability to distinguish eating patterns in general populations [15]. Turkish validation studies have supported the use of translated three-factor eating measures in obesity-related research [16].

Body image and body–self relations are also relevant because weight status is experienced through both anthropometric and psychosocial dimensions. A meta-analysis found substantially greater body dissatisfaction among people with obesity than among normal-weight individuals [17]. Internalization of body-shape ideals is strongly related to body dissatisfaction across sexes [18], and weight stigma is associated with disordered-eating cognitions and behaviors [19]. The MBSRQ provides a multidimensional assessment of appearance, fitness, health orientation, and body-area satisfaction, and its Turkish adaptation has been used to quantify overall body–self relations [20].

Mass catering workers therefore represent an occupationally specific population in which repeated food exposure, eating behavior, body evaluation, and psychosocial work conditions converge. Existing studies have generally examined excess weight, food-service employment, workplace diet, stress, or body image separately [2,3,4,5,6,7,8,9,10,11,12,13,14,17,18,19]. The present study examined associations of BMI and obesity with body–self relations, TFEQ-R18 eating behaviors, and demand-control-support characteristics among mass catering workers in Isparta, Türkiye. BMI was analyzed continuously as the primary outcome, while obesity was evaluated as a secondary binary outcome. Higher BMI was hypothesized to be associated with less favorable body–self relations and greater uncontrolled eating after adjustment for demographic, health, meal-skipping, and occupational characteristics.

## 2. Materials and Methods

### 2.1. Study Design and Reporting Framework

This observational cross-sectional study was conducted among adults employed in mass catering services in Isparta, Türkiye. Data collection took place between February and December 2025. The manuscript was structured in accordance with the Strengthening the Reporting of Observational Studies in Epidemiology (STROBE) recommendations for cross-sectional studies, including explicit reporting of setting, participant selection, variables, measurement, study size, missing data, confounding control, and sensitivity analyses [21].

### 2.2. Participants and Study Size

Eligible participants were volunteer mass catering workers aged 18–58 years who were actively employed during the study period. Eligible workers were selected using random sampling and recruited through face-to-face contact. The planning-stage sample-size target was at least 180 participants at a 95% confidence level. The final analytic sample comprised 452 participants. A sensitivity power calculation was performed for the primary linear regression using an F test for linear multiple regression ($R^{2}$ deviation from zero). With N=452, 14 predictor degrees of freedom, α=0.05, and 80% power, the minimum detectable Cohen $f^{2}$ was 0.0417. The calculation was conducted in R version 4.4 using the pwrpackage version 1.3.0. Participants with completed core anthropometric and questionnaire data were retained in the analytic dataset. The observed age range in the final sample was 18–51 years.

### 2.3. Data Collection and Covariates

Data were collected using a structured face-to-face questionnaire administered by the research team. Demographic and health variables included age, sex, marital status, physician-diagnosed health problems, vitamin-mineral supplement use during the previous 12 months, and meal-skipping behavior. Physician-diagnosed health problems were represented in the analytic dataset as a binary no/yes variable; condition-specific diagnoses were not available as separate analysis variables. Meal skipping was classified as no, sometimes, or yes. Participants who reported sometimes or regularly skipping meals were asked which meal was most commonly skipped and the principal reason. These follow-up fields were not applicable to non-skippers and were therefore treated as structurally missing rather than imputed.

### 2.4. Anthropometric Assessment and BMI Definition

Body weight was measured with a calibrated electronic scale (Medical Scale DR-Mod 85), and standing height was measured with a portable stadiometer. Participants were measured without shoes and in light clothing. BMI was calculated as weight in kilograms divided by height in meters squared. Weight status was classified using standard adult thresholds: underweight < 18.5 kg/m^2^, normal weight 18.5–24.9 kg/m^2^, overweight 25.0–29.9 kg/m^2^, and obesity ≥30.0 kg/m^2^ [22]. For descriptive presentation, all four categories were retained. Only three participants were underweight; inferential group comparisons therefore combined underweight and normal-weight participants as BMI < 25.0 kg/m^2^, producing three analysis groups (BMI < 25.0, overweight, and obesity). A comparable grouping strategy has been used in food-handler research when the underweight category is sparse [3].

### 2.5. Multidimensional Body–Self Relations Questionnaire

Body–self relations were evaluated with the 57-item Multidimensional Body–Self Relations Questionnaire (MBSRQ). The study version contains seven domains covering appearance evaluation and orientation, fitness evaluation and orientation, health evaluation and orientation, and body-areas satisfaction. Items are scored on a five-point Likert scale. Items 12, 13, 14, 25, 26, 27, 29, 30, 31, 33, 35, 37, 39, 40, and 41 are reverse-coded. The total score ranges from 57 to 285, and a mean item score is obtained by dividing the total score by 57; higher values indicate more favorable body–self relations. The Turkish adaptation reported high internal consistency (Cronbach’s alpha = 0.94) [20]. The mean item score was used in the primary analyses because it retains the original scale direction while remaining directly interpretable on the 1–5 response metric.

### 2.6. Three-Factor Eating Questionnaire-Revised 18

Eating behavior was assessed with the TFEQ-R18, which measures uncontrolled eating, cognitive restraint, and emotional eating [15]. The instrument contains 18 items: nine for uncontrolled eating, six for cognitive restraint, and three for emotional eating. Items are scored using the study’s four-point response format, and subscale scores are transformed to a 0–100 metric using the theoretical minimum and range of each subscale. Higher scores represent greater expression of the corresponding eating behavior. Turkish studies have supported the validity of three-factor eating measures for obesity-related behavioral assessment [16,23,24]. No global TFEQ score was used in the present analyses because the revised instrument represents distinct behavioral dimensions rather than a single unidimensional construct.

### 2.7. Swedish Demand-Control-Support Questionnaire

Occupational psychosocial characteristics were assessed with the Turkish version of the Swedish Demand-Control-Support Questionnaire [25]. The 17-item instrument includes job demand (5 items), job control (6 items), and social support (6 items). Job control consists of skill discretion (4 items) and decision authority (2 items). Responses are scored from 1 to 4 and summed within domains. Job control is the sum of skill discretion and decision authority. A job–stress ratio is calculated as job demand divided by job control; higher demand together with lower control reflects greater job strain within the demand-control model [13,25]. The primary adjusted models included job demand, job control, and social support as separate covariates because this parameterization retains the distinct occupational domains and avoids mechanically combining a ratio with its numerator and denominator.

### 2.8. Bias, Missing Data, and Quality Checks

Several procedures were used to reduce avoidable measurement and analytic bias. Anthropometric outcomes were based on measured rather than self-reported height and weight. The same questionnaire structure and scoring rules were applied to all participants. Formula audits confirmed that recalculated BMI and questionnaire-derived scores matched the analysis file. Missingness was examined before modeling. Core variables used in the primary models were complete, while skipped-meal and meal-skipping-reason fields were missing only when the conditional question did not apply. No statistical imputation was performed. Duplicate-record checks identified no duplicated participants. Influential observations were examined with Cook’s distance and were retained in primary analyses; models excluding flagged observations were used only as sensitivity analyses.

### 2.9. Statistical Analysis

Continuous variables were summarized with mean, standard deviation, median, and interquartile range, and categorical variables with counts and percentages. Between-group comparisons across the three inferential BMI categories used Kruskal–Wallis tests because several behavioral scores were non-normally distributed and group sizes were unequal. Effect size was summarized with $\epsilon^{2}$. To control the false discovery rate across the family of BMI-group comparisons, *p*-values were adjusted with the Benjamini–Hochberg procedure [26]. When an overall comparison was significant, pairwise Wilcoxon tests were evaluated with Holm correction [27]. Spearman rank correlations were used for monotonic associations between BMI and continuous behavioral scores. The primary multivariable analysis modeled BMI as a continuous outcome using linear regression. Prespecified adjustment variables were age, sex, marital status, chronic-disease status, vitamin-mineral supplement use, meal-skipping category, body–self mean item score, uncontrolled eating, cognitive restraint, emotional eating, job demand, job control, and social support. HC3 heteroscedasticity-consistent standard errors were used to reduce sensitivity of inference to non-constant residual variance and leverage [28]. Model diagnostics included the Breusch–Pagan test, variance-inflation diagnostics, and Cook’s distance. Obesity (BMI ≥30.0 kg/m^2^) was examined as a secondary binary outcome using multivariable logistic regression with HC3 robust standard errors. Because obesity prevalence was high, odds ratios were supplemented by modified Poisson regression with robust variance to estimate prevalence ratios as a sensitivity analysis [29,30]. Additional sensitivity analyses excluded observations exceeding the Cook’s-distance screening threshold (4/n). Direction and magnitude of key associations were compared with the full-sample estimates. All tests were two-sided, and statistical significance was defined as *p* < 0.05 after the stated multiplicity adjustment where applicable. Analyses were conducted in R version 4.4 (R Foundation for Statistical Computing, Vienna, Austria).

### 2.10. Ethical Considerations

The study was approved by the Süleyman Demirel University Health Sciences Ethics Committee on 6 January 2025 (meeting no. 88, decision no. 23). The approved protocol was titled “Determination of the Relationship between Nutritional Status, Anthropometric Measurements, Body Perceptions, Eating Habits in Emotional States and Job Stress Levels of Individuals Working in Mass Catering Systems.” Participation was voluntary, and informed consent was obtained before data collection. Study procedures were conducted in accordance with the principles of the Declaration of Helsinki.

## 3. Results

### 3.1. Participant Characteristics and BMI Profile

**Figure 1:**
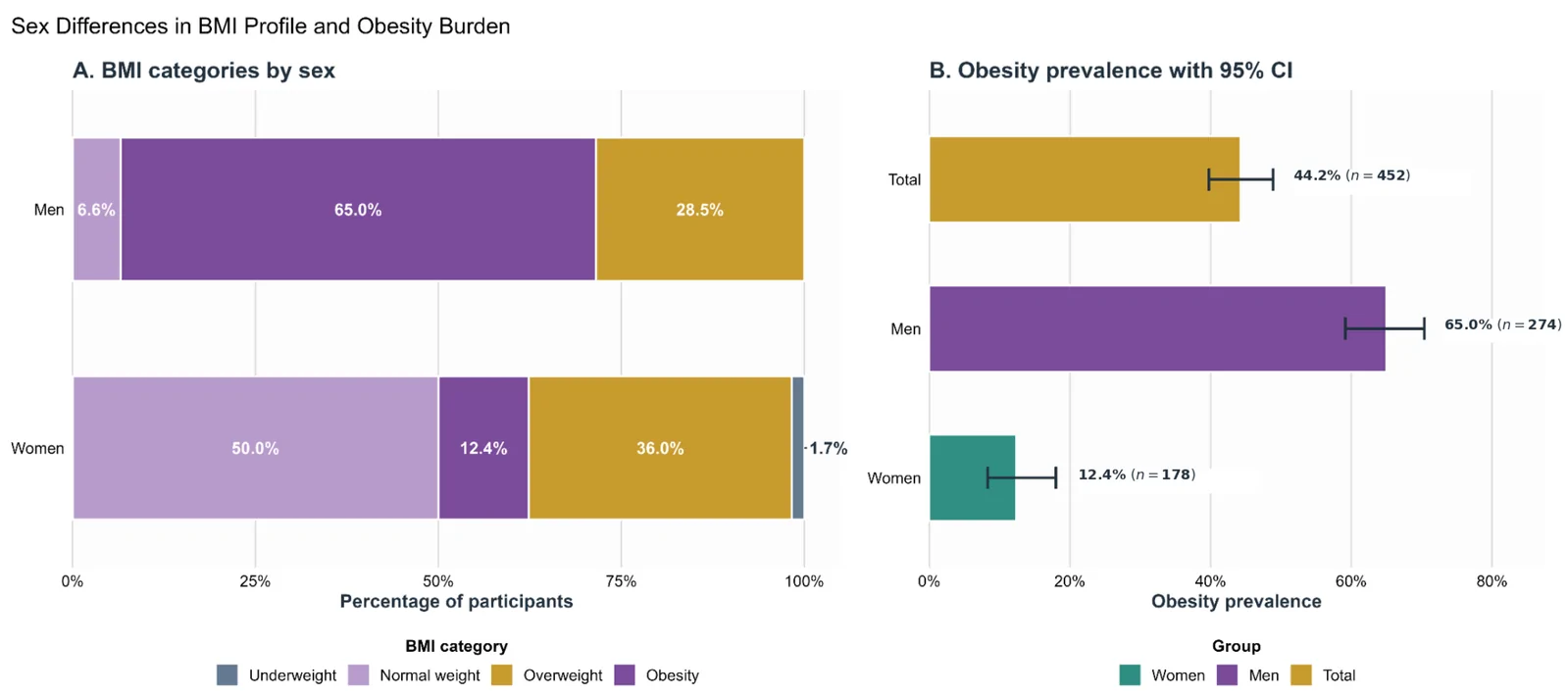
Sex differences in BMI profile and obesity burden. (**A**) BMI-category distribution by sex, showing underweight, normal weight, overweight, and obesity. (**B**) Obesity prevalence in the total sample and by sex, with 95% confidence intervals.

The analytic sample included 452 workers. Participant characteristics by BMI category are summarized in Table 1. Mean age was 34.69 ± 7.84 years, mean body weight was 80.63 ± 16.95 kg, mean height was 168.06 ± 8.28 cm, and mean BMI was 28.38 ± 4.66 kg/m^2^. Women represented 39.4% of the sample and men 60.6%. Most participants were married (86.7%), 20.1% reported a physician-diagnosed health problem, and 48.2% reported vitamin-mineral supplement use during the previous 12 months. Meal skipping was reported regularly by 34.1% and sometimes by 32.3%; 33.6% reported no meal skipping. Only three participants (0.7%) were underweight. Normal weight, overweight, and obesity accounted for 23.7%, 31.4%, and 44.2% of the sample, respectively; combined overweight and obesity prevalence was 75.7%. Obesity prevalence differed markedly by sex: 12.4% (22/178) among women and 65.0% (178/274) among men. The full four-category BMI distribution is shown in Figure 1A; obesity prevalence estimates are shown in Figure 1B.

### 3.2. Body–Self, Eating-Behavior, and Occupational Scores Across BMI Groups

**Figure 2:**
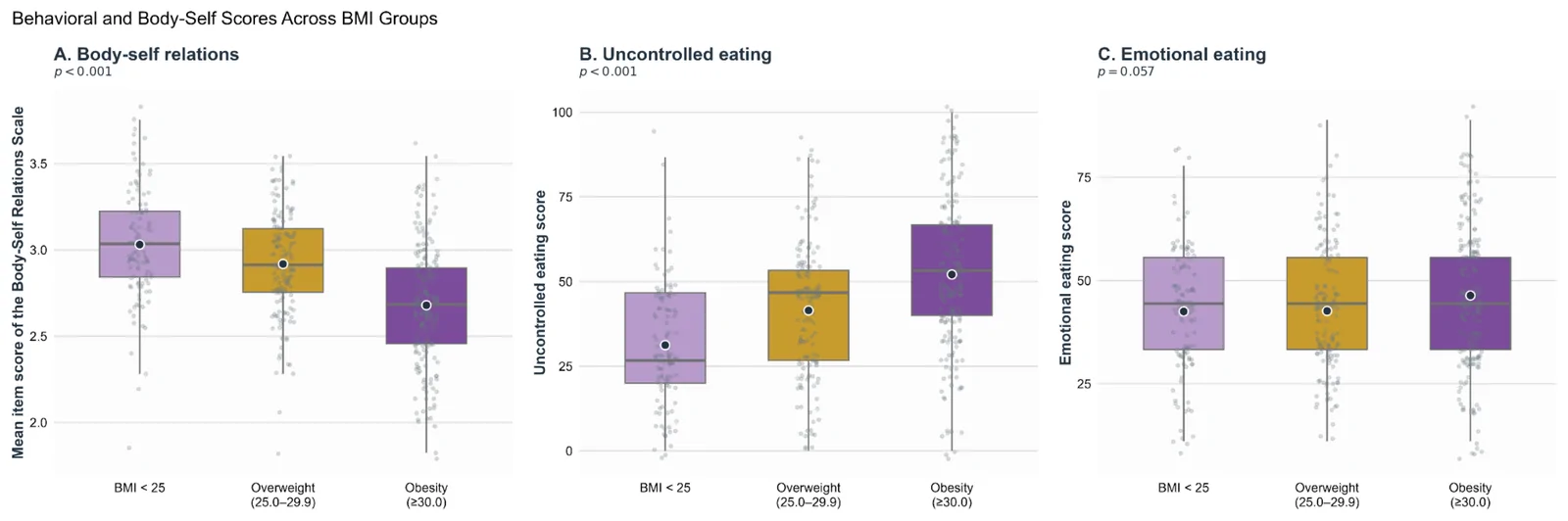
Distributions across inferential BMI groups. (**A**) Body–self relations; (**B**) uncontrolled eating; (**C**) emotional eating. Boxes show the interquartile range with the median line; small points represent individual participants, and outlined points indicate group means. The displayed *p*-values are unadjusted Kruskal–Wallis results; BH-adjusted results are reported in Table 2.

Table 2 summarizes the selected behavioral and occupational scores across BMI groups. Because the underweight group contained only three participants, inferential comparisons used BMI < 25.0 kg/m^2^ (*n* = 110), overweight (*n* = 142), and obesity (*n* = 200). The body–self mean item score declined progressively from 3.03 in the BMI < 25 group to 2.92 in the overweight group and 2.68 in the obesity group. The overall difference was large relative to the other tested domains (*H* = 82.10, $\epsilon^{2}$ = 0.178, Benjamini–Hochberg-adjusted *p* < 0.001). All three pairwise contrasts remained significant after Holm correction. Uncontrolled eating increased across the same categories, from a mean of 31.22 in the BMI < 25 group to 41.46 in the overweight group and 52.07 in the obesity group (*H* = 67.78, $\epsilon^{2}$ = 0.147, adjusted *p* < 0.001); all pairwise contrasts were significant. Cognitive restraint did not differ across BMI groups (adjusted *p* = 0.864). Emotional eating did not differ significantly across BMI groups before or after false-discovery-rate adjustment (raw *p* = 0.057; BH-adjusted *p* = 0.103). Job control and the job–stress ratio had nominal overall *p*-values of 0.034 and 0.017, respectively, but neither remained below 0.05 after Benjamini–Hochberg correction (adjusted *p* = 0.077 and 0.052). The distributions of body–self relations, uncontrolled eating, and emotional eating are shown in Figure 2A–C, respectively.

### 3.3. Correlations with BMI

**Figure 3:**
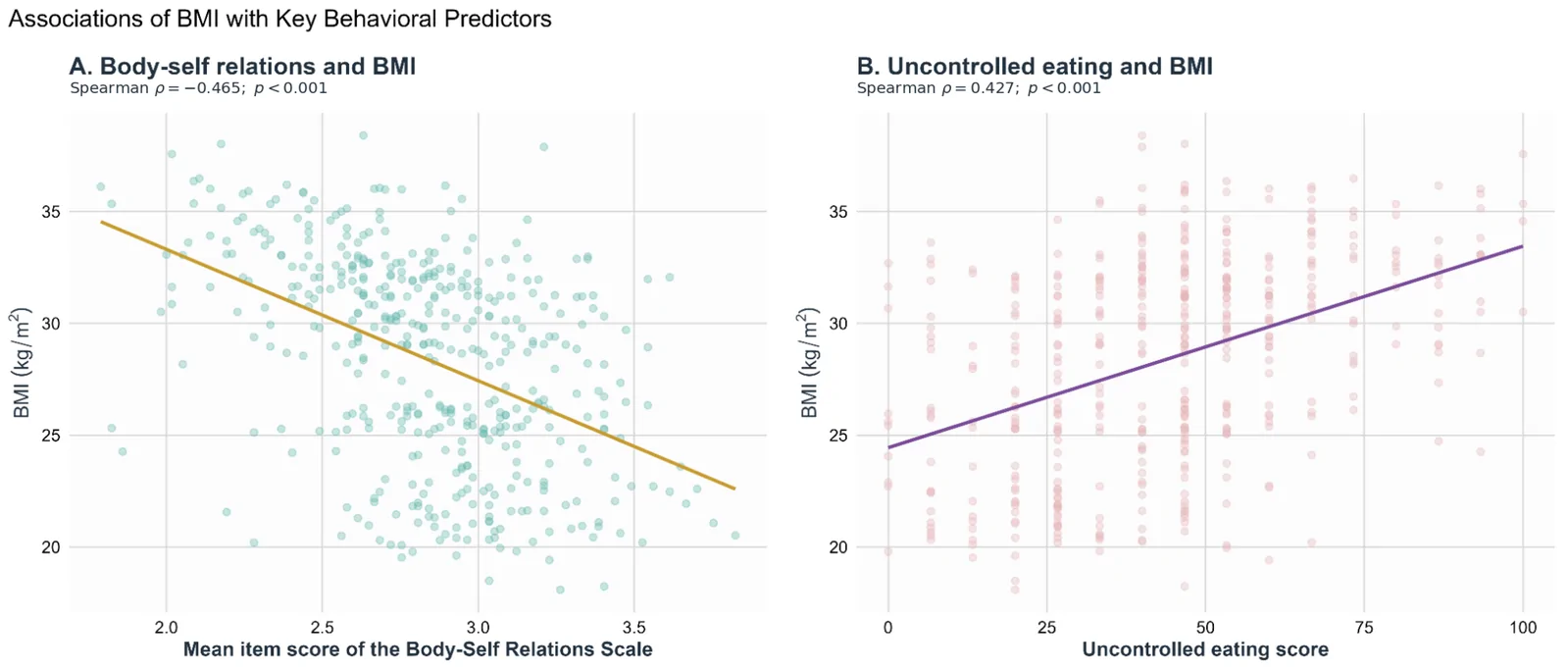
BMI associations. (**A**) Body–self relations and BMI; (**B**) uncontrolled eating and BMI. Panel annotations report Spearman correlations and two-sided *p*-values; fitted lines illustrate association direction.

BMI showed a moderate negative correlation with body–self relations (Spearman ρ = −0.465, *p* < 0.001) and a moderate positive correlation with uncontrolled eating (ρ = 0.427, *p* < 0.001). Correlations with cognitive restraint (ρ = −0.024), emotional eating (ρ = 0.111), job demand (ρ = −0.026), job control (ρ = 0.098), social support (ρ = −0.072), and the job–stress ratio (ρ = −0.106) were weak. The two principal behavioral associations are displayed in Figure 3A,B.

### 3.4. Primary Multivariable Model for BMI

The key adjusted associations from the primary linear model are presented in Table 3. The fully adjusted linear model explained approximately half of the observed variation in BMI ($R^{2}$ = 0.493; adjusted $R^{2}$ = 0.476). Residual heteroscedasticity was not detected by the Breusch–Pagan test (*p* = 0.110), and variance-inflation diagnostics did not indicate material multicollinearity. HC3 robust standard errors were retained as prespecified. body–self relations and uncontrolled eating remained independently associated with BMI after adjustment. A one-point increase in the MBSRQ mean item score was associated with a 2.82 kg/m^2^ lower BMI (β = −2.816, 95% CI −3.855 to −1.778; *p* < 0.001). Each one-point increase in uncontrolled eating on the 0–100 scale was associated with a 0.0347 kg/m^2^ higher BMI (95% CI 0.0190 to 0.0503; *p* < 0.001), equivalent to approximately 0.35 kg/m^2^ per 10 points. Male sex was associated with a 4.56 kg/m^2^ higher adjusted BMI (95% CI 3.74 to 5.38; *p* < 0.001). Job control showed a smaller positive association (β = 0.110 per point, 95% CI 0.029 to 0.191; *p* = 0.008). Age, marital status, chronic-disease status, vitamin-mineral supplement use, meal skipping, cognitive restraint, emotional eating, job demand, and social support were not statistically significant in the primary model.

The model included age, sex, marital status, chronic-disease status, vitamin-mineral supplement use, meal-skipping category, body–self relations, uncontrolled eating, cognitive restraint, emotional eating, job demand, job control, and social support. HC3 robust standard errors are reported.

### 3.5. Secondary Obesity Models and Sensitivity Analyses

Table 4 presents the adjusted odds ratios and prevalence ratios from the secondary obesity models. In secondary logistic regression, male sex was associated with higher odds of obesity (OR = 8.49, 95% CI 4.62 to 15.60), while higher body–self relations were associated with lower odds (OR = 0.173 per one-point MBSRQ mean item score, 95% CI 0.078 to 0.384). Uncontrolled eating (OR = 1.016 per point, 95% CI 1.002 to 1.029) and job control (OR = 1.068 per point, 95% CI 1.005 to 1.135) were positively associated with obesity. Modified Poisson sensitivity analysis gave directionally consistent estimates for body–self relations (PR = 0.546, 95% CI 0.420 to 0.710), uncontrolled eating (PR = 1.0048 per point, 95% CI 1.0005 to 1.0092), and job control (PR = 1.027, 95% CI 1.005 to 1.050). The male prevalence ratio was 4.02 (95% CI 2.61 to 6.19). Excluding 37 observations flagged in the binary-outcome influence screen retained the direction and statistical evidence for body–self relations, uncontrolled eating, and job control, but the magnitude of the sex estimate increased substantially. Influential-observation exclusion in the continuous-BMI model produced comparatively stable estimates for the principal body–self and uncontrolled-eating coefficients.

## 4. Discussion

Among 452 mass catering workers, overweight and obesity were common, with 75.7% of participants classified above the normal-weight range and 44.2% meeting the BMI criterion for obesity. Body–self relations and uncontrolled eating showed the clearest behavioral associations with BMI across group comparisons, correlations, and adjusted models. Occupational variables contributed smaller associations, with job control retained in the primary multivariable model. This pattern integrates behavioral, body-related, and occupational dimensions within a workforce whose daily activities occur in a food-rich environment.

The prevalence pattern is consistent with evidence showing that employment in food-service settings does not confer protection from excess weight. Studies of food-service workers have reported substantial levels of overweight and obesity, and longer employment in foodservice has been associated with excess weight [2,3]. Food insecurity has also been linked with overweight among government-backed restaurant workers [31]. Workplace cafeteria and food-environment interventions indicate that availability, pricing, product placement, labeling, and point-of-purchase cues can alter food selection and dietary behavior [32,33,34]. In mass catering, these environmental exposures are integral to the occupation because food is prepared, handled, served, and often consumed within the same setting.

Body–self relations showed a strong inverse association with BMI. Mean MBSRQ scores declined from the BMI <25 group through overweight to obesity, and the association remained after multivariable adjustment. Body image is responsive to changes occurring during weight-management interventions [35], while internalized weight stigma is associated with negative affect and disordered-eating cognitions [19,36,37,38]. The present pattern reflects the close coexistence of body-related self-evaluation and weight status in this occupational sample. Cross-sectional measurement limits temporal ordering, so the observed association encompasses both the experience of body weight and the psychological evaluation of the body.

Uncontrolled eating increased stepwise across BMI groups and remained positively associated with BMI in the adjusted model. Intervention research has documented changes in uncontrolled eating, emotional eating, and cognitive restraint during weight-loss programs [39], while behavior-change approaches targeting self-regulation and eating-related responses have also been associated with changes in emotional eating and weight [40]. Within the present workforce, uncontrolled eating was the eating-behavior dimension most consistently aligned with BMI, supporting its relevance as a behavioral marker in occupational obesity research.

Emotional eating showed a different pattern. The BMI-group comparison was not statistically significant before false-discovery-rate adjustment (raw *p* = 0.057) or after adjustment (BH-adjusted *p* = 0.103), and emotional eating was not independently associated with BMI in the primary model. Workplace research has linked occupational stress and adverse work environments with emotional eating [41]. The weaker association in the present sample indicates that emotional eating and uncontrolled eating capture distinct dimensions of eating behavior and are appropriately interpreted separately.

Occupational psychosocial associations were comparatively modest. The primary model included job demand, job control, and social support as separate covariates, and job control showed a positive adjusted association with BMI. Job demand and social support were not independently associated with BMI. The job–stress ratio was examined in the BMI-group comparisons, where its raw group difference was attenuated after BH adjustment, and it was not included as a covariate in the primary multivariable specification. Evidence on occupational stress and adiposity remains heterogeneous across worker populations [10,11,12], while rotating shift schedules have been associated with altered meal timing and greater nighttime eating [42]. The positive job-control coefficient in this sample may also reflect occupational role, skill use, and task structure captured within the control construct. A review of randomized workplace wellness trials found heterogeneous results for stress, food choice, and weight management, underscoring the need for consistent measures and study designs [43].

Meal skipping was common across the sample but was not independently associated with BMI in the primary model. Observational evidence has linked breakfast skipping with overweight and obesity [44], whereas randomized-trial evidence has not shown a consistent anthropometric benefit from routinely eating breakfast [45,46]. The meal-skipping responses in this study included practical reasons such as lack of prepared food, lack of habit, low appetite, weight-control intentions, and time constraints, placing meal timing within the organization of the workday as well as within individual eating behavior.

A marked sex difference was present in the BMI distribution. Obesity prevalence was 65.0% among men and 12.4% among women, and male sex had the largest adjusted coefficient in the continuous-BMI model. The magnitude of the sex association varied more in binary-outcome influence analyses than in the continuous-BMI model. This pattern points to sex-related differences within the sampled workforce that may involve job roles, physical workload, work schedules, and eating opportunities not captured in the present covariate set.

The findings also connect with workplace prevention research. Food-environment interventions in cafeterias, hospitals, and other institutional settings have modified dietary choices through changes in availability, placement, labeling, pricing, and purchasing cues [32,33,47]. Behavioral support can complement environmental change, particularly when eating responses and body-related experiences are part of the same occupational context [34,39,40]. The inverse body–self association also aligns with evidence linking stigmatizing weight experiences to adverse psychological and eating-related outcomes [19,36,37,38].

Study strengths include measured anthropometry, a final sample substantially larger than the planning-stage minimum, standardized questionnaire scoring, explicit control of multiple comparisons, robust standard errors, and influence sensitivity analyses. Continuous BMI was retained as the primary outcome, preserving information across the full observed weight distribution. The three underweight participants were retained descriptively and combined with normal-weight participants only for inferential BMI-group comparisons.

The cross-sectional design limits temporal interpretation of associations among body–self relations, eating behaviors, occupational characteristics, and BMI. Behavioral and psychosocial measures were self-reported and remain subject to recall and social-desirability effects. The study was conducted in mass catering services in one Turkish region, which limits geographic generalizability. Condition-specific chronic diagnoses were not available as separate analytic variables, so health status was adjusted as a binary physician-diagnosed disease indicator. Physical activity, shift schedule, sleep, socioeconomic position, detailed dietary intake, and job-role information were also unavailable for the final adjusted model. BMI does not distinguish fat mass from lean mass. The secondary obesity models were more sensitive to influential observations than the continuous-BMI model, particularly for the magnitude of the sex association.

Taken together, the findings identify body–self relations and uncontrolled eating as the most consistent behavioral correlates of BMI in this mass catering workforce. Their associations were evident across descriptive, correlational, adjusted, and sensitivity analyses, while occupational psychosocial measures showed smaller and more variable relationships. Longitudinal occupational studies incorporating body composition, work schedules, detailed food exposure, body–self measures, and eating-behavior domains can further characterize how these factors develop over time.

## 5. Conclusions

Among mass catering workers in Isparta, overweight and obesity were highly prevalent. Higher BMI was consistently associated with less favorable body–self relations and greater uncontrolled eating across BMI-group comparisons, correlations, and adjusted analyses. Occupational psychosocial variables showed smaller associations, with job control retained in the primary BMI model while job demand and social support were not independently associated. These findings provide an occupationally specific characterization of obesity-related variation in a workforce continuously exposed to food preparation and service environments and place eating behavior and body-related self-evaluation alongside workplace characteristics in the study of employee weight status.

## Figures and Tables

**Table 1:** Participant characteristics by BMI category.

Characteristic	Underweight (n=3)	Normal Weight (n=107)	Overweight (n=142)	Obesity (n=200)	Total (n=452)
Age, years	31.67 ± 2.08	33.56 ± 7.98	34.35 ± 7.82	35.59 ± 7.75	34.69 ± 7.84
Body weight, kg	51.20 ± 0.69	60.78 ± 7.45	76.36 ± 8.87	94.73 ± 11.33	80.63 ± 16.95
Height, cm	167.40 ± 2.02	165.88 ± 7.18	167.04 ± 8.29	169.97 ± 8.50	168.06 ± 8.28
BMI, kg/m^2^	18.27 ± 0.20	22.02 ± 1.43	27.31 ± 1.68	32.69 ± 1.82	28.38 ± 4.66
Sex: Women	3 (100.0%)	89 (83.2%)	64 (45.1%)	22 (11.0%)	178 (39.4%)
Sex: Men	0 (0.0%)	18 (16.8%)	78 (54.9%)	178 (89.0%)	274 (60.6%)
Marital status: Married	3 (100.0%)	88 (82.2%)	121 (85.2%)	180 (90.0%)	392 (86.7%)
Marital status: Single	0 (0.0%)	19 (17.8%)	21 (14.8%)	20 (10.0%)	60 (13.3%)
Physician-diagnosed health problem: No	3 (100.0%)	87 (81.3%)	115 (81.0%)	156 (78.0%)	361 (79.9%)
Physician-diagnosed health problem: Yes	0 (0.0%)	20 (18.7%)	27 (19.0%)	44 (22.0%)	91 (20.1%)
Vitamin/mineral supplement use: No	3 (100.0%)	48 (44.9%)	71 (50.0%)	112 (56.0%)	234 (51.8%)
Vitamin/mineral supplement use: Yes	0 (0.0%)	59 (55.1%)	71 (50.0%)	88 (44.0%)	218 (48.2%)
Meal skipping: No	2 (66.7%)	33 (30.8%)	53 (37.3%)	64 (32.0%)	152 (33.6%)
Meal skipping: Sometimes	1 (33.3%)	38 (35.5%)	41 (28.9%)	66 (33.0%)	146 (32.3%)
Meal skipping: Yes	0 (0.0%)	36 (33.6%)	48 (33.8%)	70 (35.0%)	154 (34.1%)

Values are mean ± SD for continuous variables and *n* (%) for categorical variables; percentages are calculated within BMI categories. The underweight category was retained for descriptive presentation and combined with normal weight only in the three-group inferential analyses.

**Table 2:** Selected behavioral and occupational scores across inferential BMI groups.

Variable	BMI <25 (n=110)	Overweight (n=142)	Obesity (n=200)	Raw *p*	BH-Adjusted *p*
Body–self relations, mean item score	3.03 ± 0.32	2.92 ± 0.31	2.68 ± 0.34	<0.001	<0.001
Uncontrolled eating, 0–100	31.22 ± 17.79	41.46 ± 20.76	52.07 ± 21.88	<0.001	<0.001
Emotional eating, 0–100	42.51 ± 14.60	42.63 ± 15.66	46.38 ± 16.86	0.057	0.103
Job control	13.84 ± 4.13	14.64 ± 4.03	15.06 ± 4.19	0.034	0.077
Job–stress ratio	0.845 ± 0.316	0.775 ± 0.356	0.764 ± 0.340	0.017	0.052

Values are mean ± SD. Raw *p*-values are from Kruskal–Wallis tests; BH-adjusted *p*-values use the Benjamini–Hochberg procedure across the prespecified family of nine BMI-group comparisons. Inferential grouping combined the three underweight participants with the normal-weight category. BH, Benjamini–Hochberg.

**Table 3:** Key adjusted associations with BMI in the primary HC3 linear model.

Variable	Adjusted β	95% CI	*p*-Value
Male sex (vs. women)	4.562	3.744 to 5.380	<0.001
Body–self relations, per 1-point MBSRQ mean item score	−2.816	−3.855 to −1.778	<0.001
Uncontrolled eating, per 1 point (0–100)	0.0347	0.0190 to 0.0503	<0.001
Job control, per 1 point	0.1098	0.0286 to 0.1911	0.008

**Table 4:** Secondary obesity associations: logistic odds ratios and modified Poisson prevalence ratios.

Variable	Adjusted OR	95% CI	*p*	Adjusted PR	95% CI	*p*
Male sex	8.494	4.624 to 15.602	<0.001	4.018	2.608 to 6.190	<0.001
body–self relations	0.173	0.078 to 0.384	<0.001	0.546	0.420 to 0.710	<0.001
Uncontrolled eating	1.0155	1.0024 to 1.0289	0.020	1.0048	1.0005 to 1.0092	0.030
Job control	1.068	1.005 to 1.135	0.033	1.027	1.005 to 1.050	0.017

OR, odds ratio; PR, prevalence ratio. Both models used robust standard errors and the same principal adjustment structure as the BMI model.

## Data Availability

The data supporting the findings of this study are available from the corresponding author on reasonable request, subject to ethical and privacy restrictions applicable to participant-level data.
